# Latent Potential of Multifunctional Selenium Nanoparticles in Neurological Diseases and Altered Gut Microbiota

**DOI:** 10.3390/ma16020699

**Published:** 2023-01-11

**Authors:** Hajra Ashraf, Davide Cossu, Stefano Ruberto, Marta Noli, Seyedesomaye Jasemi, Elena Rita Simula, Leonardo A. Sechi

**Affiliations:** 1 Department of Biomedical Sciences, University of Sassari, 07100 Sassari, Italy; 2 Complex Structure of Microbiology and Virology, AOU Sassari, 07100 Sassari, Italy

**Keywords:** selenium, nanoparticles, neurological diseases, gut microbiota

## Abstract

Neurological diseases remain a major concern due to the high world mortality rate and the absence of appropriate therapies to cross the blood–brain barrier (BBB). Therefore, the major focus is on the development of such strategies that not only enhance the efficacy of drugs but also increase their permeability in the BBB. Currently, nano-scale materials seem to be an appropriate approach to treating neurological diseases based on their drug-loading capacity, reduced toxicity, targeted delivery, and enhanced therapeutic effect. Selenium (Se) is an essential micronutrient and has been of remarkable interest owing to its essential role in the physiological activity of the nervous system, i.e., signal transmission, memory, coordination, and locomotor activity. A deficiency of Se leads to various neurological diseases such as Parkinson’s disease, epilepsy, and Alzheimer’s disease. Therefore, owing to the neuroprotective role of Se (selenium) nanoparticles (SeNPs) are of particular interest to treat neurological diseases. To date, many studies investigate the role of altered microbiota with neurological diseases; thus, the current review focused not only on the recent advancement in the field of nanotechnology, considering SeNPs to cure neurological diseases, but also on investigating the potential role of SeNPs in altered microbiota.

## 1. Introduction

Neurological diseases are regarded as the world’s leading cause of disability and mortality, and they account for 12% of global deaths. The most common neurological diseases include Alzheimer’s disease, Parkinson’s disease, and multiple sclerosis [1].

The central nervous system comprises the brain and spinal cord, which play an important role in neurological diseases. According to the body’s function and regulation, the CNS has three predominant barriers, i.e., the blood–brain barrier (BBB), the cerebrospinal fluid–blood barrier (the avascular arachnoid epithelium), and the blood–cerebrospinal fluid barrier (the choroid plexus epithelium). Owing to these naturally existing barriers, particularly the BBB, the treatment of neurological diseases through drug delivery into the CNS is challenging [2]. However, there are some FDA-approved drugs that are currently used for neurological disease treatment (Table 1).

Currently, there is no effective therapy for many neurological diseases. Scientists and technologists from multidisciplinary fields, i.e., from behavior to the molecular level, have carried out research in multiple directions, but a truly interdisciplinary way of treatment has not yet been explored. The ultimate consequence of this is that many pathological disorders involving the central nervous system (CNS) remain untreated.

Nanoparticles (NPs) represent a promising approach in the treatment of neurodegenerative diseases, specifically Parkinson’s and Alzheimer’s disease (REF) [3,4]. Drug delivery through nanosized particles not only crosses the blood–brain barrier but also makes for target-specific delivery. Moreover, numerous benefits are associated with NPs to treat the CNS, i.e., high biological and chemical stability, ability to be administered by various routes, large surface-to-volume ratio, and feasibility to incorporate both hydrophobic and hydrophilic drugs [5,6].

Selenium (Se), being an important trace element in the body, showed remarkable health benefits, i.e., improving the immune system [7], securing the nervous system’s physiological activity [8], and combating oxidative damage caused by free radical species [9]. As an integral component of selenoproteins, Se has an essential role in the fundamental functioning of the CNS [10]. Therefore, a deficiency of Se contributes to the pathogenesis of various neuropathological and neurodegenerative diseases. Se supplementation has numerous beneficial impacts on neurological diseases. However, Se has a narrow range between toxic and beneficial doses. The Expert Group on Vitamins and Minerals (EVM) recommended that the daily dose of Se should be 60 μg for women and 70 μg for men [11,12], a dose above 400 μg is considered toxic and leads to a disorder known as selenosis. Owing to their incredible health benefits, Se (selenium) nanoparticles (SeNPs) gained worldwide attention due to their wide application in the field of therapeutics. SeNPs have lower toxicity, higher efficiency to resist free radical species, and acceptable bioavailability in comparison to inorganic selenium. Moreover, based on the experimental data, the toxicity of SeNPs is classified as lower than that of other organic and inorganic compounds such as selenate, selenite, and selenomethionine. SeNPs are involved in numerous physiological and metabolic processes, such as the regulation of the immune system and the antioxidant defense system [13,14,15]. Additionally, SeNPs have a strong capability to penetrate biological cells and tissues, suggesting their potential efficiency to inhibit oxidative stress and inflammation [16,17]. Owing to these unique advantages, recently, SeNPs have snatched a lot of attention from scientists for their use in the treatment of neurological diseases.

In light of the above-mentioned discussion, the current review summarizes the potential benefits of SeNPs to treat neurological diseases. Since recent studies have investigated the role of the altered microbiota in neurological diseases, this review also provides insight into how SeNPs can regulate the altered microbiota, a crucial step in opening new perspectives on the use of SeNPs as potential pharmacotherapy.

## 2. Materials and Methods

This review is based on Google Scholar and PubMed searches using the following keywords: neurological diseases, selenium nanoparticles, and microbiota. The final search was performed in December 2022, and recent papers with high relevance were selected for the review.

## 3. Results and Discussions

### 3.1. Selenium Compounds and Their Physiological Effects

Selenium is an essential trace element that plays an important role in various physiological functions, including reactive oxygen species (ROS) control and modulation in the immune system [18]. According to the European Food Safety Authority, the recommended daily allowance (RDA) of Se is 70 µg day^−1^ for men, 60 µg/day for women [19], 65 µg day^−1^ for pregnant women, and 75 µg day^−1^ for lactating women [20,21].

The main form of Se is the Se analog of the amino acid methionine known as selenomethionine (SeMet), which is absorbed and makes an entry to the methionine pool in the body after digestion [22,23]. Selenium in the form of inorganic selenate and selenite are mostly used as supplementation. Se often plays a major role in the generation of selenoproteins that is essential for the body due to their multiplex roles, i.e., protein folding, control over thyroid hormone metabolism, redox signaling, etc. [20].

Se is also known to have antibacterial, antiviral, antifungal, and antitumor properties. In addition, various studies confirmed Se’s role in thyroid, cardiovascular, and neurological diseases [19,20,24]. Se adequate amount supports the immune system by enhancing the activity of natural killer (NK) cells and the proliferation of T cells against pathogens and cancer cells, also enhancing the efficacy of vaccines [25,26]. It also contributed to the reduction of risk associated with various inflammation-related diseases like rheumatoid arthritis [27]. Se maintains ROS production and enhances DNA stability while decreasing the renal and hepatic side effects of chemotherapeutic drugs [18].

#### 3.1.1. Selenium Bioavailability, Metabolism, and Physiological Functions

The bioavailability of Se depends upon the food consumed [28], being more prevalent in animal products than in vegetables. The content of Se is more influenced by the source of the animal and also its species, i.e., fish have elevated levels of Se. SeMet is abundant in both animals and plants, whereas selenocysteine is mostly present in animals. The principal form of selenium in the body is SeMet, as it enters the Se pool directly [23,29].

Under physiological conditions, all forms of Se have an absorption rate of 70–90%, except selenite, which has a lower absorption rate of 60%. In addition, food processing also influences bioavailability, as proteins are more easily digestible at higher temperatures and Se release and bioavailability become more efficient. Due to synergistic, additive, and antagonistic interactions, total carbohydrate, fat, protein, and fiber contents also influenced Se bioavailability [30,31].

Se metabolism occurs mainly in the liver as it is responsible for selonoprotein synthesis and excretion via various selenometabolites. Mostly Se is excreted through urine, while some is significantly excreted through feces [30].

#### 3.1.2. Se Potential Therapeutic Impact

Various studies confirmed the immunomodulatory and anti-inflammatory role of Se and its supplementation has been demonstrated to cure various anti-inflammatory diseases, i.e., chronic lymphedema, Crohn’s disease, asthma, and chronic lymphedema [24,32]. Se is also known to be effective against cancer as it decreases ROS production and prevents gene dysfunction and DNA damage generated by oxidative stress in the body. It is also used as a chemotherapeutic and radiotherapy adjuvant, as its pro-oxidant effects are more effective on malignant cells than on healthy cells. [24,33].

Deficiency of Se correlates with various bacterial, parasitic, and viral infections, which show the influence of Se on the function of the immune system [25,34] as HIV, H1N1, influenza, West Nile virus infection, etc. [29]. Se supplementation has also been proven to be favorable for the treatment of numerous bacterial infections such as *Mycobacterium tuberculosis*, *Helicobacter pylori*, *Escherichia coli*, etc. Instead of provoking an immune response against the poliovirus and influenza A vaccinations, Se supplementation has also corresponded to antiparasitic properties against *Heligmosomoides bakeri* and *Trypanosoma cruzi* [25,34].

The potential role of Se in cardiovascular diseases has been revealed by numerous studies due to its protection against excessive platelet aggregation and oxidative damage, which ultimately stop the pathologies of cardiovascular diseases, i.e., heart hypertrophy, atherosclerosis, congestive failure, and hypertension [24,29,35]. Due to the regulatory effect of selenoproteins on the insulin signaling cascade, Se is also associated with the prevention of type 2 diabetes. Reduction in insulin resistance is shown to be due to selenoproteins, as they diminish pancreatic insulin production and indirectly thioredoxin reductases (TR) lower insulin resistance. However, some studies depict a higher association of Se supplementations with a greater risk of type 2 diabetes, so the role of Se in diabetes is not yet clear [36,37].

**Figure 1:**
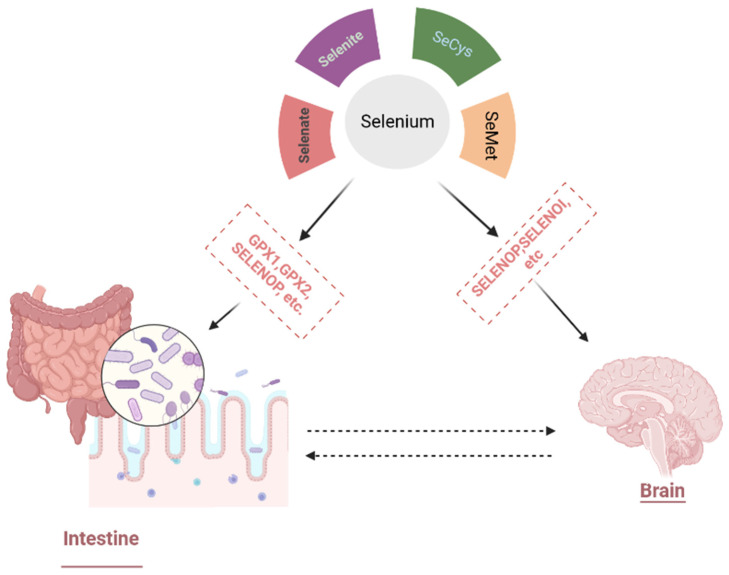
Different selenoproteins regulate different organs in the body. Se is mainly absorbed in the form of selenoproteins. GPX1 and GPX2 maintain the body’s health by regulating the production of reactive oxygen species (ROS). SELENOP normally acts as a plasma transporter in numerous organs, while DIO1 affects thyroid hormone activity. SELENOI, on the other hand, is involved in managing the nervous system, and a deficiency of SELENOI results in the emergence of neurodegenerative diseases.

Selenium is present in glandular and gray matter regions of the brain and contributes to various dopaminergic and neurotransmission pathways; hence, it is also used as a potential biomarker in various neurological diseases, i.e., Alzheimer’s, epilepsy, and Parkinson’s diseases [24,38]. The antioxidant neuroprotective function of Se creates a strong impact on the hyperphosphorylation of the tau protein, cytoskeleton assembly regulation, Aβ deposition attenuation, and the tendency to bind with neurotoxic metals, which constitutes its ability to have a potential role in the development of Alzheimer’s disease. Various selenoproteins were also studied to protect dopaminergic neurons, strengthening the neuroprotective role of Se against Parkinson’s disease [39,40]. Considering the low level of Se in the brain, the applications of Se will only be beneficial for patients who have severe Se deficiency, lower selenoprotein production, or mutations in genes associated with the delivery of Se [39]. The main Se potential in neurological diseases is described in Figure 1.

### 3.2. Preparation and Characterization Methods of Se Nanoparticles (SeNPs)

#### SeNP Production Methods

**Figure 2:**
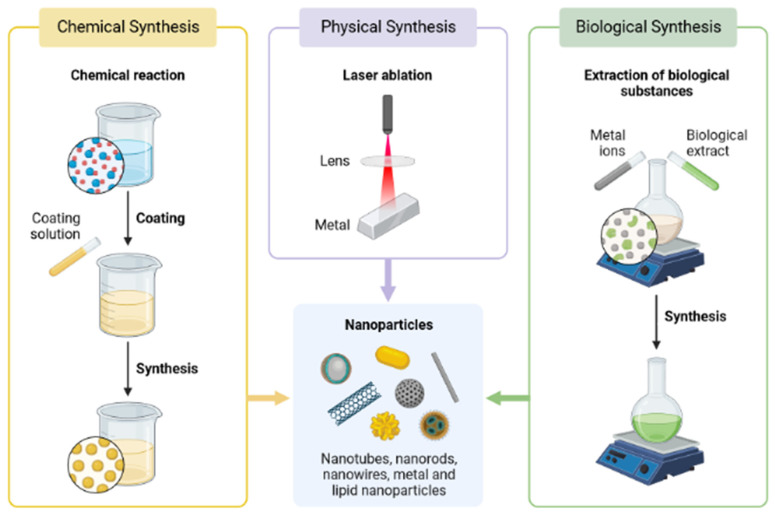
Nanoparticles Methods of Production.

Se can be synthesized from three common methods of obtaining nanoparticles: physical, chemical, and biological methods (Figure 2). Since chemical methods involved the use of high temperatures, dangerous chemicals, and an acidic pH for the catalytic reduction of ionic selenium, this represented a less safe method for the synthesis of Se nanoparticles (SeNPs) [41,42]. Physical methods such as electrodeposition techniques, phyto-thermal-associated synthesis, and microwave synthesis are less common than chemical methods. The third and most effective method used nowadays is the biological method, which uses algae, yeast, fungi, and plants as biological catalysts for the production of nanoparticles. The biological method is advantageous over the other two methods due to its lower cost, fast growth rate of microorganisms and plants, lower toxicity, common procedures for culturing, the nonexistence of severe extreme conditions, and eco-friendly production of nanoparticles (Table 2) [43,44,45,46].

The biosynthesis of SeNPs has been conducted using various plant extracts, i.e., *Cinnamomum zeylanicum* bark, fresh citrus and lemon fruits [47], Aloe vera leaf extracts, *Dillenia indica* [48], *Vitis vinifera* [49], *Prunus amygdalus* leaf [50], *Allium sativum* [51], etc. The main benefit of using plant extracts is that plant’s secondary metabolites themselves act as natural reductant and stabilizer agents in an eco-friendly approach.

Due to the biological activities of selenium, SeNPs are widely used for various biomedicinal applications, for example, in the treatment of neurological diseases and diabetes as an antiviral, antibacterial, anti-apoptotic, and anti-inflammatory drug, and for the effective delivery of selective drugs into the tissues.

It is important to determine the physical characteristics of nanoparticles because the shape and size of nanoparticles affect their activity on cells and tissues. For example, Se nanowires have higher photoconductivity, while spherical-shaped SeNPs have been proven to have higher biological activities [21]. The antioxidant properties of nanoparticles also depend on their size: SeNPs have been shown to scavenge free radicals in a size-dependent manner (5–200 nm) [52]. The functionalization of NPs with other substances also depends upon the shape and size of NPs, i.e., the effectiveness of chitosan as an antioxidant and antitumor agent firmly depends upon SeNPS characteristics [16,53]. The synthesis method influences the shape and size of NPs and, consequently, their medicinal properties. There are different forms of SeNPSs, such as rod-like, hexagonally flowered, nanowires, nanotubes, nanoneedles, and nanorods. The spherically shaped SeNPs are more commonly used for pharmacological and biological purposes [54].

### 3.3. Role of SeNP in Neurodegenerative Diseases

Se, being a principal trace element in humans and animals, plays a remarkable role in regulating the standard physiological functions of the brain. It also has a neuroprotective role, and some selenoproteins also participate in the protection against neurodegenerative diseases. Studies proved that the metabolism of Se in the brain is different from that in other body organs, as Se remains preserved in the brain in the case of Se deficiency [55,56,57]. Currently, SeNPs’ role in brain diseases has been studied because neurons are more prone to be damaged by oxidative stress due to several reasons, such as a low level of antioxidant enzymes, a high consumption of oxygen, and occupancy of the high level of polyunsaturated fats [58,59,60].

#### 3.3.1. Alzheimer’s Disease and SeNPs

One of the main factors in the pathogenesis of neurodegenerative diseases is oxidative stress. Numerous natural antioxidants are used as treatments, but the hurdle is their limited accuracy [56,57]. Therefore, the focus is now on the synthesis of nanoparticles that have greater antioxidant potential. Various studies showed that nanoparticles more often act as an oxidizing agent and may cause damage to neurons, decreasing the cognitive functions of Alzheimer patients’ brains [61,62,63,64,65]. Despite that, several studies pointed out that SeNPs in Alzheimer’s disease prevent the aggregation of amyloid-β (Aβ) protein and also can cross the BBB [66,67]. It has been demonstrated that SeNPs coated with epigallocatechin-3-gallate and peptide B6 had a similar effect [68]. Xianbo Zhoub et al. determined that cysteine enantiomer modified SeNPs (abbreviated as D/LSeNPs) demonstrated a strong impact on the aggregation of Aβ in the presence of metal ions, i.e., Cu^2+^ and Zn^2+^. These SeNPs modified by the chelating agent can prevent Aβ fibril formation by blocking metal ion binding sites and by binding with Aβ. Modified SeNPs are more effective in protecting the cell because of their effective absorption by PC12 cells, protection from oxidative stress, and potential to maintain cellular redox potential [69].

A considerable therapeutic promise in Alzheimer’s disease is the inhibition of amyloid β (Aβ) aggregation. Although, the non-selective disposition of drugs and BBB put a major hurdle in achieving this. A study conducted by Licong Yang et al. demonstrated that the conjugation of the targeted peptide with SeNPs acts as dual-functional NPs that not only cross the BBB but also inhibit the aggregation of Aβ [70].

Similarly, in another study conducted by Dongdong Sun et al., it was found that SeNPs coated with the chelating agent were effective in preventing Aβ aggregation, memory impairment, and ameliorating cognition [71].

Nevertheless, the current focus is on the synthesis of nanoparticles based on natural resources for the cure of AD [72], as resveratrol (Res)-polyphenol, which is mainly found in plants, has an antioxidant and especially a neuroprotective effect [73,74]. Thus, the synthesis of SeNPs with Res coating enhanced the antiaggregatory and antioxidant potency of reversatol, which was demonstrated on PC12 cells of the adrenal medulla of rats [75]. The potency of ResSeNPs to bind with Aβ42 and block the Cu^2+^ binding that leads to cell death by damaging the cell membrane has been demonstrated [75].

#### 3.3.2. SeNPs and Parkinson’s Disease

The second most progressive neurodegenerative disease is Parkinson’s disease, which has the main characteristics of muscle rigidity, dyskinesia with tremors, postural instability, and bradykinesia [76,77,78,79]. Although the pathophysiology of Parkinson’s disease is not yet clear, oxidative stress is regarded as one of the prime pathological markers of PD as it results in neuronal damage and ultimately death [80,81]. Yue Dong et al. evaluated the antioxidant and therapeutic potential of glycine-SeNPs. For the study of Parkinson’s disease MPTP (1-methyl-4-phenyl-1,2,3,6-tetrahydropyridine) is considered a potential neurotoxin. Two animal group models were designed with and without MPTP to check the neuroprotective effect of glycine-SeNPs. Results depicted that glycine-SeNPs decreased the MDA level and increased GSH-PX activity and SOD activity, thus influencing a neuroprotective effect in comparison to MPTP-induced PD rats [82].

### 3.4. Selenium Nanoparticles and Gut–Brain Axis

About 2500 years ago, Hippocrates stated that the gut was responsible for the beginning of all diseases. With time, this statement gains a lot of support from the ongoing research on animal models and humans. The gut is regarded as the home of a diverse and complex ecosystem of trillions of microorganisms that include yeasts, bacteria, viruses, protozoa, and archaea [83]. The human gut microbiota is considered a unique entity that is shaped by lifestyle and diet, and as a result, the physiology of the host is shaped by microorganisms [84,85]. Host and gut microbiome symbiotic relationships start when embryonic development is shaped by maternal microbiota and initiate gut microbiota colonization during birth and development [86,87,88]. The microbiota influenced the maturation of the neural, immune, and endocrine systems and played a remarkable role in cognitive and postnatal brain development [89,90,91].

#### Methods in the Study of the Microbiota

**Figure 3:**
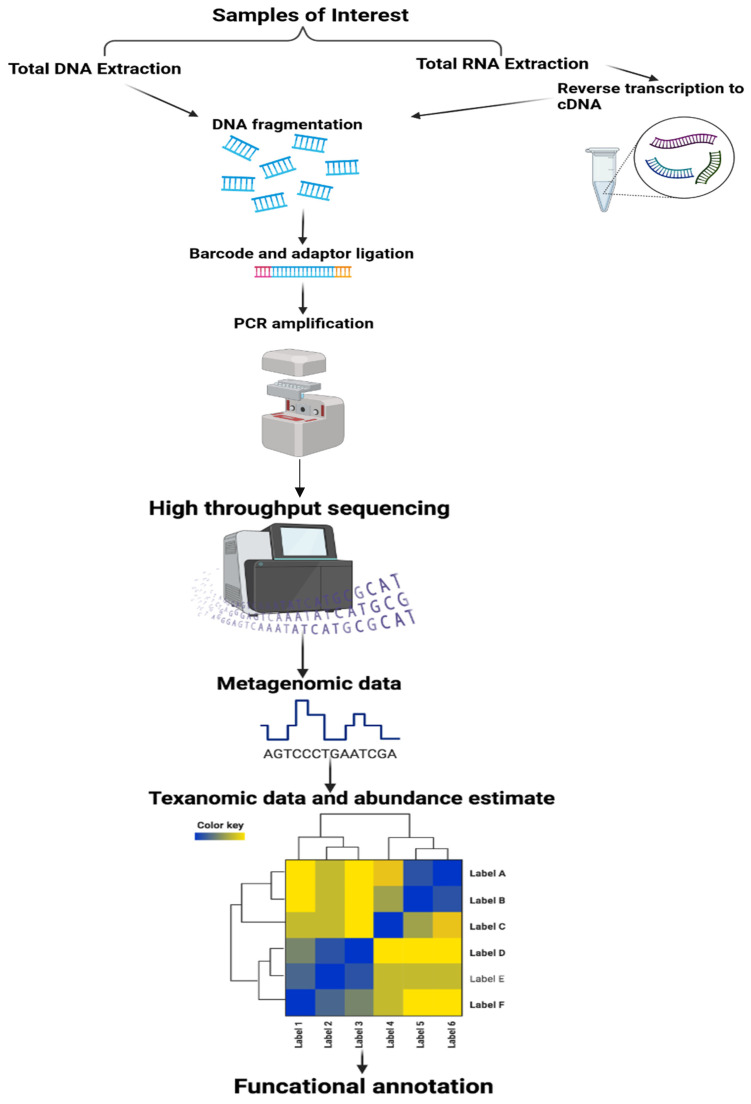
Overview of key steps involved in metagenome study.

High-throughput DNA sequencing technologies have made possible the detailed study of the microbiome. The two techniques that have largely been used to study the microbiome are based on whole metagenome sequencing and 16S ribosomal RNA gene sequencing (Figure 3). The initial steps for both methodologies involved the isolation of microbial cells from host cells, DNA extraction, and amplification using a random primer (for metagenomics) or gene-specific primers (16s rRNA).

The gene that encodes 16s rRNA is a unique identifier of closely related and individual species because it contains both highly conserved and hypervariable regions. The 16sRNA gene identified the bacterial species in the sample either by comparing it with the reference genome or by clustered de novo. This approach uses quantitative measures to describe species’ evenness, diversity, and relative abundance of specific groups of closely related species. In the metagenomic approach, unbiased sequencing of DNA is conducted for all the microbial species present in the sample [92,93].

### 3.5. Gut Microbiota and Neurodegenerative Diseases

Microorganisms living in the gastrointestinal tract (GI) have gained prime interest in studies of their role in neurological diseases. The GI tract is extremely vascularized, having an enriched lymphatic system tract, and is animated by a multiplex enteric nervous system, which is renowned as “the second brain”. Thus, there are numerous access points through which luminal microbes can gain access and influence the host immune response either directly or indirectly. The diverse population of microorganisms, i.e., Firmicutes and Bacteroidetes, largely participate in the colonization of the GI tract [93,94]. The gut commensal microbes enhanced the digestion and absorption of nutrients and yielded enhanced enzymatic activity by expressing unique genes [95]. Gut microbes use compounds derived from these nutrients as a source of metabolic intermediates and energy [96]. Thus, it becomes clear that the gut microbiome has a considerable role in human physiology, and dysbiosis results in a wide range of neurological and other diseases, including diabetes and obesity.

#### 3.5.1. Parkinson’s Diseases (PD)

The pathology of the gut is a well-known marker of Parkinson’s disease. About 60–80% of patients suffered from constipation up to 20 years before the clinical onset of PD, and it is regarded as one of the earliest symptoms [97,98,99]. It is noteworthy that at the earliest stage of the disease, deposition of α-synuclein is observed even before motor pathology onset [100,101,102]. Considering these findings, it is suspected that in the gut, the pathology of PD occurs before expanding into the brain. Chandra et al. [103] conducted a study to gain insight into the role of the gut microbiome in PD. A germ-free gnotobiotic animal model is used for the study. It was observed that ASO mice growing in germ-free conditions overexpress α-synuclein as compared to colonized ASO mice. The germ-free ASO mice were then inoculated with microbial metabolites derived from carbohydrates and short-chain fatty acids, which, as a result, promoted the pathology of PD. Additionally, antibody treatment enhanced the PD burden. Appealingly recolonization of ASO mice with the microbiota of healthy donors results in improved cognitive behavior in PD mice in comparison to ASO mice recolonized with the microbiota of PD patients. Gut microbiome dysbiosis is also revealed in human PD. Compared to control, microbial species, i.e., *Ralstonia*, *Coprococcu*, *Blautia*, and *Roseburia*, are increased in PD patients, while microbial communities belonging to the *Prevotellaceae and Faecalibacterium* families are decreased in the observed stool samples. It is also observed that *Enterobacteriaceae* family abundance is also significantly associated with gait dysfunction and postural instability [104,105].

#### 3.5.2. Alzheimer’s Disease (AD)

The relationship between gut microbiota and AD pathogenesis is well understood in the animal model. Minter et al. [106] first reported the relationship of AD with microbiota. It was observed that the murine model of AD was influenced by antibiotic-induced perturbations in the gut microbiota diversity, and as a result, amyloidosis and neuroinflammation occurred. In another study, the sequencing of 16s rRNA was performed by analyzing the fecal samples of APP transgenic mice with the control, which revealed a significant gut microbiome difference between them. In germ-free transgenic APP mice, cerebral Aβ was also reduced. However, the recolonization of germ-free transgenic APP mice with the microbiota of transgenic APP mice results enhanced the level of cerebral Aβ, and this effect was less when the microbiota of wild-type mice was used [107].

#### 3.5.3. Multiple Sclerosis (MS)

The gnotobiotic mouse also has been effective in studying MS pathology’s relationship with microbiota [108]. Transgenic EAE mice grown in sterile environments experienced no diseases or markedly attenuated disease; however, colonization with MS patients’ microbiota restored the phenotype of EAE [109,110]. Further studies supported this linkage of microbiota with MS pathology, i.e., Berer k. et al. [111] observed in their study that oral administration of *Bifidobacterium animalis* and *Bacteroides fragilis* reduced the development of MS disease. The role of human gut microbiota in MS directly comes from the comparison of the microbiota of healthy controls and MS patients. One large study reported that microbial populations, i.e., *Akkermansia*, *Butyricimonas*, and *Methanobrevibacter*, are different between both MS patients and healthy controls [112]. Vicente Navarro et al. researched the linkage of gut microbiota with MS patients having active relapsing-remitting multiple sclerosis (RRMS). The results showed a difference in microbial species at *Clostridium*, *Hungatella*, *Lachnospiraceae*, *Shuttleworthia*, *Bilophila*, *Poephyromonas*, and *Ruminococcaceae* between healthy control and RRMS patients [113]. In another study, Sherein G.Elgendy et al. found that alterations in microbiota are directly linked with the exacerbation of MS. Disruption in intestinal microbiota results in the enrichment or depletion of certain bacteria that leads to MS predisposition. *Desulfovibrio*, *Firmicutes*, *Actinobacteria*, and lactic acid bacteria were higher in MS patients in comparison to healthy controls, while *Clostridium cluster IV* is comparatively lower in MS patients [114]. A new perspective on how microbiota influenced MS patients was explained by Atsushi Kadowaki et al. A study found that gut microbiota-dependent CCR9 CD4 T cells were altered in secondary progressive multiple sclerosis (SMPS), which leads to the development of SMPS [115].

### 3.6. Selenium Nanoparticles, Microbiota, and Neurodegenerative Diseases

The synergetic communication between the central nervous system and gut, mediated by gut microbiota, plays a significant role in the development of neurological diseases such as Alzheimer’s disease [116] (Table 3). Vogt et al. [117] conducted an extensive sequencing of stool samples, showing the difference between microbiome diversity in healthy controls and AD patients. At the phylum level, actinobacteria have a lower prevalence, while Firmicutes are present in abundance. Similarly, at the genus level, *Gemella*, *Blautia*, *Alistipes*, and *Phascolarctobacterium* are at a higher level in comparison to *Clostridium* and *Bifidobacterium*, which are less abundant. The difference in the microbiome between AD and healthy controls strongly suggested that altered gut microbiota are directly linked with alternations in AD neuropathology. Another study conducted by Mancuso et al. found excessive *Shigella* abundance in comparison to *Eubacterium rectale* in amyloid-positive patients [118]. Probiotics have a significant effect on modulating the gut–brain axis.

**Figure 4:**
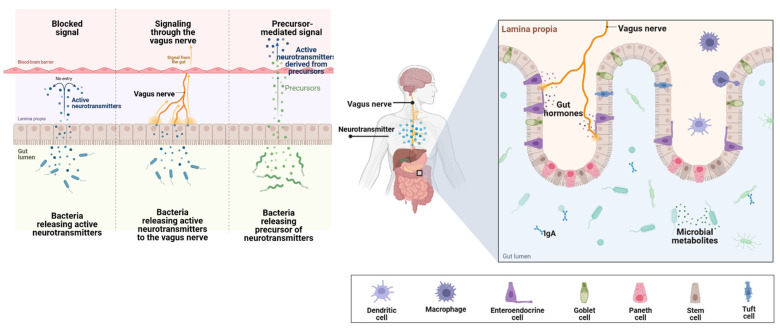
Gut-brain axis.

Additionally, microbiota dysbiosis also leads to the secretion of inflammatory-related molecules, such as lipopolysaccharide and amyloids, and causes damage to the intestinal mucosal barrier, ultimately stimulating neuroinflammation and microglia activation, which are possibly involved in the progression of neurodegeneration [119]. Enhanced permeability of the intestine causes enhanced metabolite accumulation and translocation, resulting in microbial community imbalance [120]. One of the important pattern recognition receptors that are involved in brain inflammation through the activation and release of microglia and other inflammatory factors is Toll-like receptor 4 (TLR4). TLR4 is majorly activated by lipopolysaccharide (LPS), resulting in the activation of inflammation-related signaling pathways. [121]. Hou et al. [122] demonstrated that high plasma LPS levels and intestinal permeability directly correspond with inflammatory cytokine expression in mouse brains. Therefore, high levels of LPS may cause microglia activation because of intestinal barrier dysfunction. Hence, the microbiota–gut–brain axis concept was based on the communication between the brain and the gut microbiota achieved by the enteric nervous system, the vagus nerve, the immune system, and microbial metabolites, i.e., tryptophan, proteins, and short-chain fatty acids (SCFAs) (Figure 4). Current studies investigated whether the administration of probiotics enhanced the pathophysiology of autoimmune neurological diseases, i.e., AD. Akbari et al. [123] illustrated in their study that the administration of probiotics containing *Lactobacillus casei*, *Lactobacillus fermentum*, *Lactobacillus acidophilus*, and *Bifidobacterium bifidum* had a positive effect on AD patients.

Similarly, a meta-analysis suggested that probiotics influenced the cognitive behavior of AD patients by decreasing oxidative stress and neuroinflammation levels [124]. Thus, the results strongly convinced us of the potential efficacy of probiotics in AD patients by improving cognitive dysfunction [125]. Selenium, a micronutrient, plays an important role in redox regulation because of its integration into selenoproteins. Koc E.R. et al. [126] documented a direct relationship between Se deficiency and cognitive impairment in AD patients. In another study conducted by Tamtaji et al. [127], it was demonstrated that the administration of Se in combination with multiple probiotics enhanced the metabolic profile and mini-mental state examination (MMSE) score of AD patients. Additionally, the supplementation of sodium selenite at high or super nutritional levels results in high Se uptake by the central nervous system, which significantly improves MMSE scores [128]. However, several concerns are associated with sodium selenates, which limit their implementation in the food and medicine industries, i.e., low biological activity, high toxicity, not easy absorption and utilization by the human body, and a narrow range of safe supplementation [129]. Currently, SeNPs have gained a lot of attention due to their high bioactivity, low toxicity, and high bioavailability. Moreover, based on experimentation data, Se species toxicity is ranked as selenate > selenite > selenomethionine > SeNPs.

A recent study conducted by Lei Qiao et al. [130] showed that administration of SeNPs enriched with *Lactobacillus casei* ATCC 393 averted cognitive dysfunction in AD mice through the modulation of the microbiota-gut-brain axis. ATCC 393 SeNPs minimize aggregation of amyloid beta (Aβ) protein and modulate brain-derived neurotrophic factor (BDNF) or Akt/cAMP-response element binding protein (CREB) pathways that prevent neuronal death. Additionally, SeNPs caused TAU protein hyperphosphorylation, improved cognitive dysfunction, restored gut microbiota balance, regulated immune response, and enhanced production of SCFAs, which ultimately inhibit microglia activation and protect the neuronal cells from neurotoxicity, i.e., neuroinflammation, and oxidative stress. Thus, *L. casei* ATCC 393-SeNPs may act as a safe and promising nutritional supplement to avert neurological diseases.

Licong Yang et al. [131] studied the effect of surface-modified SeNPs in Alzheimer’s disease mice. SeNPs were coated with dihydromyricetin (DMY), as it was unstable under physiological conditions, so it was further coated with chitosan (CS). To cross the blood–brain barrier, CS/DMY SeNPs were further coated with the BBB-targeted peptide Tg; thus, the resultant Tg-CS/DMY@SeNPs that easily cross the BBB inhibit the aggregation of Aβ protein and reduce the secretion of inflammatory cytokines through the NF-κB pathway. Moreover, it repairs the gut barrier and regulates the gut microbiota species, i.e., *Dubosiella*, *Bifidobacterium*, and *Desulfovibri*. Moreover, Tg-CS/DMY@SeNPs enhanced the relative abundance of *Gordonibacter*, which downregulates the NLRP3 inflammasome protein expression and decreases the serum inflammatory factor concentration. Through this, it is suggested that Tg-CS/DMY@SeNPs reduce neuroinflammation in the gut microbiota-NLRP3 inflammasome brain axis.

Moreover, Tg-CS/DMY@SeNPs enhanced the relative abundance of *Gordonibacter*, which downregulates the NLRP3 inflammasome protein expression and decreases the serum inflammatory factor concentration. Through this, it is suggested that Tg-CS/DMY@SeNPs reduce neuroinflammation in the gut microbiota-NLRP3 inflammasome brain axis.

**Figure 5:**
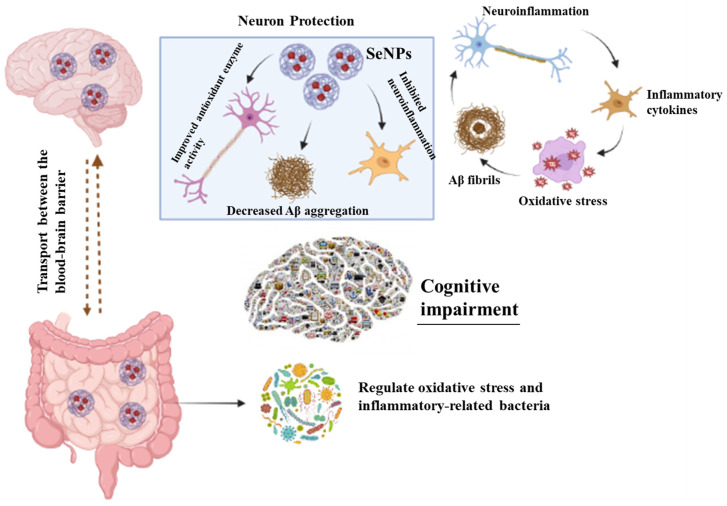
SeNPs’ mechanism of action to diminish Alzheimer’s disease-like pathogenesis.

Resveratrol (Res) has a neuroprotective effect, but it has lower bioavailability. Changjiang Li et al. [132] illustrated for the first time that oral administration of resveratrol selenium peptide nanocomposites regulated gut microbiota and reduced Aβ aggregation by diminishing Alzheimer’s disease-like pathogenesis. The mechanism of action involved binding with Aβ and decreasing aggregation, lowering ROS, and increasing antioxidant enzyme activity, activating the Akt signaling pathway that results in the downregulation of neuroinflammation, averting inflammatory-related gut bacteria and oxidative stress, and helping to overcome gut microbiota dysbiosis (Figure 5).

Thus, the abovementioned studies illustrated that functionalized SeNPs are potential drug candidates for treating neurological diseases, particularly Alzheimer’s disease.

**Table 3:** Effect of SeNPs on neurological diseases and microbiota.

Nanomaterials	Average Size	Experimental Model	Dose	Exposure Time	Administration Way	Gut Microbiota Alteration	Effects to Host	References
TGN-Res@SeNPs	14 nm	AD model mice	50 mg/kg b.w.	16 weeks	Oral gavage	1. Decrease of Desulfovibrio, Candidatus_Saccharimonas, Ruminococcaceae_UCG-014, Lachnoclostridium, Enterorhabdus, and Faecalibaculum; 2. Increase of Lachnospiraceae_NK4A136_ group, Alistipes, Odoribacter, Helicobacter and Rikenella	Alleviation of Alzheimer’s disease-like pathogenesis	[132]
Biogenic SeNPs	170.5 to 182.5 nm	SD rats	0.5, 1.0 or 2.0 mg/kg	-	Administered by gavage	1. Protected the integrity of the spinal cord 2. Decreased the expression of several inflammatory factors 3. Enhanced the production of M2-type macrophages by regulating their polarization, indicating a suppressed inflammatory response	Improve the disturbed microenvironment and promote nerve regeneration	[133]
DMY@SeNPs	46.30 nm	APP/PS1 mice	50 mg/kg body weight	16 weeks	Oral gravage	Regulate the population of inflammatory-related gut microbiota such as Bifidobacterium, Dubosiella, and Desulfovibrio	Ameliorate neuroinflammation through the gut microbiota-NLRP3 inflammasome-brain axis	[131]

## 4. Conclusions

In conclusion, the main aim of this review was to organize the latest data on the pharmacotherapeutic potential of SeNPs to treat neurodegenerative diseases. In addition, the well-studied role of microbiota in neurological diseases was also presented. To the best of our knowledge, this is the first-ever study that mentioned the role of SeNPs in treating both neurodegenerative diseases and altered microbiota at the same time. Though this study has illustrated that SeNPs could be a potential hallmark in neurological disease treatment. Moreover, the data presented in this study will help the researchers to quickly navigate the current research on SeNPs and their therapeutic potential in treating neurological diseases that are linked with altered microbiota. This review will also open new doors of research for scientists to find the potential of SeNPs to treat microbiota-related diseases and to overcome some major challenges associated with nanomaterial synthesis, i.e., the difficulty of assessing safety and effectiveness, the lack of specialized equipment for efficient and high-quality nanomaterial synthesis. Nevertheless, the treatment of neurological diseases, which is regarded as an uphill battle, could be easily overcome if multimodal agents are actively practiced with the help of nanotechnology.

## Figures and Tables

**Table 1:** FDA approved drugs for neurological diseases.

FDA-Approved Drugs for Neurological Diseases			
Drug Name	Approval	Disease	Indications
Briumvi	28 December 2022	Multiple sclerosis (MS)	BRIUMVI is a CD20-directed cytolytic antibody indicated for the treatment of relapsing forms of multiple sclerosis (MS)
Relyvrio	29 September 2022	Amyotrophic lateral sclerosis (ALS)	RELYVRIO is indicated for the treatment of amyotrophic lateral sclerosis (ALS) in adults.
Aduhelm	7 June 2021	Alzheimer’s disease	To treat Alzheimer’s disease
Suvorexant	29 January 2020	Mild-to-moderate Alzheimer’s disease (AD)	Insomnia characterized by difficulties with sleep onset and/or sleep maintenance
18F-Fluortaucipir	28 May 2020	Alzheimer’s disease (AD)	Evaluation of tau neurofibrillary tangle (NFT) density and distribution with positron-emission tomography
Ozanimod	25 March 2020	Multiple sclerosis (MS)	Relapsing multiple sclerosis (MS), including clinically isolated syndrome (CIS) and active secondary progressive MS (aSPMS) in adults
Inebulizumab	12 June 2020	neuromyelitis optica spectrum disorder (NMOSD)	Antiaquaporin-4 positive (AQP4)^+^ neuromyelitis optica spectrum disorder (NMOSD)
Satralizumab	16 August 2020	neuromyelitis optica spectrum disorder (NMOSD)	Antiaquaporin-4 positive (AQP4)^+^ neuromyelitis optica spectrum disorder (NMOSD)
Ofatumumab	20 August 2020	Multiple sclerosis (MS)	Relapsing forms of multiple sclerosis (MS), including clinically isolated syndrome (CIS) and active secondary progressive MS (aSPMS) in adults

**Table 2:** Comparative evaluation of SeNPs production methods.

Method of Production	Materials	Characteristics	Advantages	Disadvantages
Chemical Method	Inorganic Se (i.e., selenate or selenite) reduction by a reducing agent. Use of a capping agent for stabilization of nanoparticles.	Characteristics of NPs depend upon stabilizing agents.	Simple method without the need for technological instruments and biological incubation.	Use of harmful chemicals that make it a less environment-friendly method.
Physical Method	Usage of physically based methods, i.e., laser ablation, heating, etc., to induce changes in inorganic Se in the presence of the stabilizing agent.	Characteristics of NPs depend upon stabilizing agents. Small-sized nanoparticles production.	Environment-friendly process. Rapid reaction. Less energy spent.	Specific instrument necessities.
Biological Method	Use of biological agent as a stabilizing and reducing agent for inorganic selenium.	The characteristics of NPs depend upon biological organisms, i.e., plants, fungi, and yeast.	Environment-friendly process. No need of extra stabilizing agent as biological organisms itself acts as both reducing and stabilizing agent.	Need for optimization of several steps and processes in order to obtain NPs.

## Data Availability

Not applicable.
